# Supplementary material for: Functional correlates of immediate early gene expression in mouse visual cortex
Source: Peer Community J. Author manuscript; Available in PMC 2023 Apr 21. (PMC7614465; doi:10.24072/pcjournal.156)
Supplement: Supplementary Figures [file EMS174132-supplement-Supplementary_Figures.pdf]

Supplementary Figures

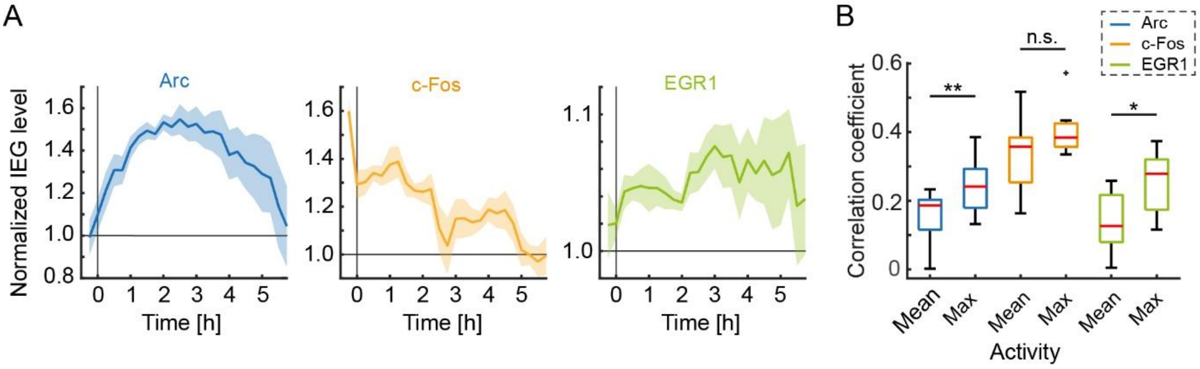

Figure S1. Time course of IEG expression during the imaging paradigm and correlation of IEG expression with mean and maximum neuronal activity. Related to Figure 1.

(**A**) Time course of normalized IEG expression levels following 24 h dark adaptation and 15 min visual stimulation at time 0. Shading indicates SEM over neurons.

(**B**) Correlation coefficient of mean and maximum activity (average across or peak within a recording session, respectively) with IEG expression 3.5 h after stimulation or recording onset (Arc: 11 mice, c-Fos: 9 mice, EGR1: 8 mice). Box whisker plot: red line indicates median, box marks 25th to 75th percentiles and whiskers extended to the next most extreme datapoint within a range of 1.5 times the interquartile distance (rank sum test, Arc: $p = 0.0086$, c-Fos: $p = 0.1359$, EGR1: $p = 0.0207$).

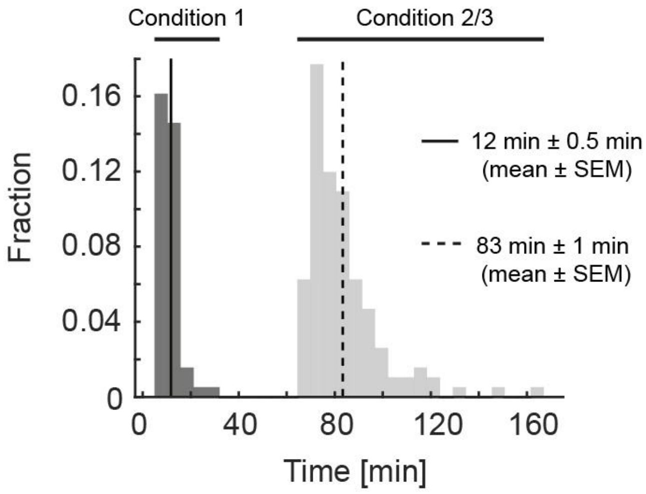

Figure S2. Duration of recording sessions. Related to Figure 2.
Histogram of the durations of the recording sessions. On average, one recording session lasted for approximately 12 min during condition 1 (solid line) and, due to the addition of closed-loop, open-loop, and grating stimulation phases, 83 min during conditions 2 and 3 (dashed line).
